# Causal effects of gut microbiota on risk of overactive bladder symptoms: a two-sample Mendelian randomization study

**DOI:** 10.3389/fmicb.2024.1459634

**Published:** 2024-08-23

**Authors:** Chaodong Shen, Mengjie Fang, Xiaolong Zhang, Zhirong Zhu, Jiajian Chen, Guiliang Tang

**Affiliations:** Department of Urology, Shaoxing People's Hospital, Shaoxing Hospital of Zhejiang University, Shaoxing, China

**Keywords:** overactive bladder, urinary incontinence, gut microbiota, Mendelian randomization, causal relationship

## Abstract

**Background:**

Clinical observations indicate a correlation between the gut microbiota and overactive bladder (OAB) symptoms. Nevertheless, the causal relationship and mechanisms between gut microbiota and OAB symptoms remain elusive.

**Methods:**

Two-sample Mendelian randomization (MR) analyses were performed to assess the association between gut microbiota and OAB symptoms, including urinary incontinence (UI). Data were obtained from the MiBioGen International Consortium genome-wide association studies (GWAS) dataset and the IEU GWAS database. The inverse variance weighted method was used as the primary approach in the MR analysis, with the weighted median, MR-Egger, and weighted mode methods as supplementary approaches. Sensitivity analyses were employed to assess potential violations of the MR assumptions.

**Results:**

Our analysis identified seven gut bacterial taxa with a causal relationship to OAB and nine gut bacterial taxa associated with UI. Genera *Eubacteriumfissicatenumgroup*, *LachnospiraceaeNK4A136group*, and *Romboutsia* were identified as protective factors against OAB, while genera *Barnesiella*, *FamilyXIIIAD3011group*, *Odoribacter,* and *RuminococcaceaeUCG005* were associated with an increased risk of OAB. A higher abundance of the genus *Coprococcus3*, order Burkholderiales, and phylum Verrucomicrobia predicted a lower risk of UI. Conversely, the class Mollicutes, genus *Ruminococcus gauvreauii* group, order Mollicutes RF9, and phylum Firmicutes and Tenericutes were positively correlated with UI risk. The sensitivity analysis excluded the influence of potential heterogeneity and horizontal pleiotropy.

**Conclusion:**

This study revealed a causal relationship between gut microbiota and OAB symptoms, providing new insights and a theoretical foundation to identify biomarkers and therapeutic targets for patients with OAB symptoms.

## Introduction

Overactive bladder (OAB) is clinically diagnosed based on the presence of troublesome lower urinary tract symptoms. The International Continence Society defines OAB as the presence of “urinary urgency, usually accompanied by frequency and nocturia, with or without urgency urinary incontinence (UUI), in the absence of urinary tract infection or other obvious pathology” (Abrams et al., 2002). Previous studies conducted in different countries have reported OAB prevalence rates varying between 2.1 and 16.6% (Stewart et al., 2003; Homma et al., 2005; Irwin et al., 2006). OAB symptoms significantly impact patients’ quality of life, especially in patients with symptoms of urinary incontinence (UI); however, the precise mechanisms remain incompletely understood. A widely accepted theory explaining the pathophysiology of OAB involves detrusor overactivity (Brading, 2006). Patients with OAB are treated with anti-cholinergic drugs and β3 agonists. However, some patients still experience symptoms despite treatment, highlighting that the mechanisms underlying OAB remain poorly understood (Gormley et al., 2012). Consequently, exploring the pathophysiological mechanisms leading to OAB formation is essential. This exploration is vital for developing innovative biological targeted therapy for pharmacological treatment.

The human gastrointestinal tract harbors numerous bacteria in a healthy state, forming the largest microbiota ecosystem in the body, known as the gut microbiome, which participates in and influences the metabolism of substances and energy (Gomes et al., 2018). Increasing research has indicated that the gut microbiome is associated with the occurrence and progression of autoimmune diseases, metabolic syndromes, and cancer. It is also intimately linked to certain diseases of the urinary system, including urinary system tumors, urolithiasis, benign prostatic hyperplasia, and urinary tract infection (Jiao et al., 2020; Magruder et al., 2020; Matsushita et al., 2021 Li L. Y. et al., 2022; Luo et al., 2023;). The concepts of the gut-prostate, gut-bladder, and gut-kidney axes have garnered increasing attention (Yang et al., 2018; Salazar et al., 2022; Fujita et al., 2023). Previous research has identified a relationship between the microbiota and OAB symptoms, particularly UUI, which may be crucial in preventing, diagnosing, and treating OAB (Antunes-Lopes and Cruz, 2019; Holland et al., 2020). Approximately 64% of the bacterial species in the urinary microbiota overlap with those found in the intestine, suggesting an intestinal origin (Holland et al., 2020). A longitudinal study has suggested that the gut microbiota may be linked to the risk of OAB symptom progression, and the underlying mechanism may involve neural crosstalk between the bladder and gastrointestinal tract via parasympathetic and sympathetic pathways (Okuyama et al., 2022). However, the existence of a clear causal relationship between the gut microbiota and OAB remains uncertain due to insufficient clinical evidence.

Mendelian randomization (MR) leverages single nucleotide polymorphisms (SNPs) and genome-wide association studies (GWAS) to establish causal relationships and quantify effect sizes (Emdin et al., 2017). MR research mitigates the influence of reverse causation and minimizes confounding factors, thereby enhancing the reliability and validity of findings (Bowden and Holmes, 2019). Given that the gut microbiota does not alter an individual’s DNA sequence (Zheng et al., 2017), MR is a valuable tool to rigorously investigate the association between OAB symptoms and gut microbiota.

This study used the latest available large-scale GWAS summary statistics for MR analysis to identify the potential causal relationship between gut microbiota and OAB symptoms. It offered new perspectives for further mechanistic research and novel bio-targeted therapy, enhancing the overall quality of life for patients suffering from OAB symptoms.

## Materials and methods

### Study design

**Figure 1:**
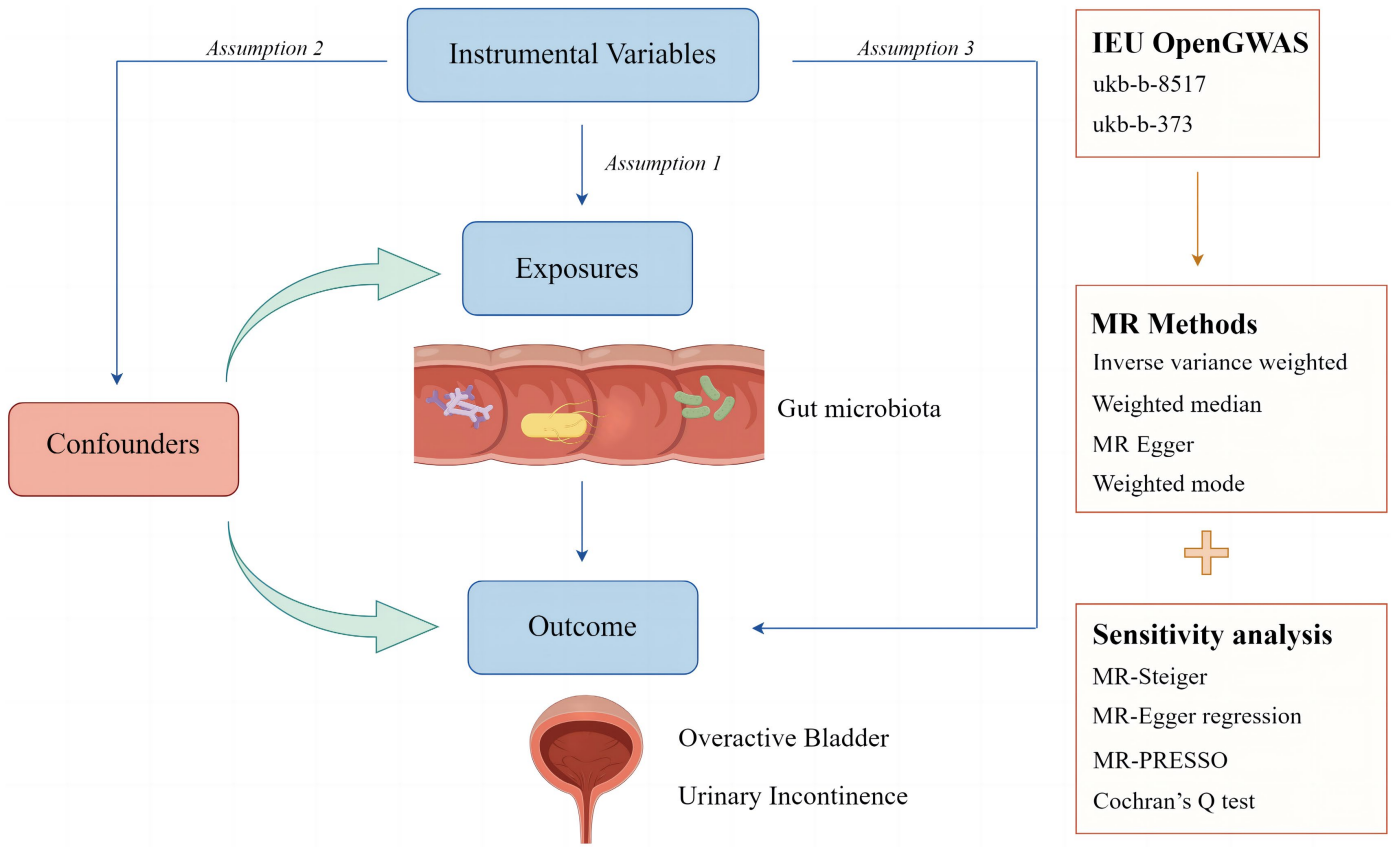
Study design and assumptions of Mendelian randomization analysis. Assumption 1: instrumental variables (IVs) are associated with the exposure of interest; Assumption 2: IVs are independent of any confounders in univariate MR; Assumption 3: IVs affect the outcome only through the exposure.

In this study, two-sample MR analyses were conducted to determine the relationship between 196 gut microbiota and OAB symptoms, including UI. No additional ethical approval was needed because all the data used were published in a public database, and the original studies from which the summary statistics were derived had obtained proper ethical approval and informed consent. The analytic process was conducted per the Strengthening the Reporting of Observational Studies in Epidemiology using MR guidelines (Skrivankova et al., 2021). The following three assumptions must be satisfied for MR studies (Labrecque and Swanson, 2018): (I) instrumental variables (IVs) are associated with the exposure of interest; (II) IVs are independent of any confounders in univariate MR; (III) IVs affect the outcome only through exposure. Figure 1 illustrates the study design and MR assumptions in this study.

### Data sources for the exposure

The MiBioGen consortium conducted a large-scale GWAS to examine the relationship between human genetic variation and the gut microbiome (Kurilshikov et al., 2021). This comprehensive study integrated the gut microbiome data from 18,340 participants across 24 cohorts from the United States, Canada, Israel, Germany, Sweden, Finland, and the United Kingdom, with the majority being of European descent (*n* = 13,266). In this study, data from the MiBioGen consortium encompassing 196 gut microbiome taxa (9 phyla, 16 classes, 20 orders, 33 families, and 119 genera) were used as exposure variables, excluding 15 unknown microbiome taxa (3 unknown families and 12 unknown genera).

### Data sources for the outcome

The SNPs associated with OAB and UI were downloaded from the IEU OpenGWAS project database. For OAB, the phenotype “Bladder: Calcified/Contracted/Overactive” (GWAS ID: ukb-b^−373^) (Zhang et al., 2024), with a sample size of 463,010 and 9,851,867 SNPs, was selected. The summary data for UI were derived from the GWAS phenotype “Urinary frequency/Incontinence” (GWAS ID: ukb-b^−8,517^) (Zhang et al., 2024), with a sample size of 462,933 and 9,851,867 SNPs. Notably, both databases were produced using a GWAS pipeline with phesant-derived variables from the UK Biobank. All participants had similar genetic backgrounds, and each individual was of European ancestry.

### Selection of IVs

The following quality control steps were implemented to select the best IVs and ensure the validity and accuracy of the conclusions drawn regarding the causal relationship between the gut microbiome and OAB symptoms. Based on the correlation hypothesis, a threshold of *p* < 1 × 10^−5^ was used to screen for SNPs significantly associated with the gut microbiome, as they rarely reach genome-wide significance (*p* < 5 × 10^−8^) (Sanna et al., 2019). Additionally, the MR method requires that the selected IVs are not in linkage disequilibrium (LD) with each other. SNPs with an R^2^ < 0.001 and clustering distance of 10,000 kb were selected to retain independent SNPs and eliminate LD. Next, palindromic SNPs and SNPs absent in the outcome dataset were excluded to ensure that the effect of SNPs on exposure corresponded to the same allele as their effect on outcome (Hemani et al., 2018). Finally, to minimize the risk of weak instrument bias, the F-statistic for each gut microbiome-related SNP was calculated using the following formula: F = R^2^ × (n − k − 1)/[k × (1 − R^2^)]. IVs with an F-statistic <10 were defined as weak instruments and removed (Burgess and Thompson, 2011; Levin et al., 2020).

### Statistical analysis

Four main methods were used in the MR analysis: inverse-variance weighted (IVW), weighted mode, MR-Egger regression, and weighted median. Among these, IVW was the primary assessment method, assuming that IVs affect the outcome solely through the exposure of interest and do not impact any other pathways (Bowden et al., 2015). A range of methods were performed for sensitivity analysis. The MR-Egger regression intercept was used to estimate directional pleiotropy (*p* < 0.05 indicated directional pleiotropy) (Bowden et al., 2015). The MR-Pleiotropy Residual Sum and Outlier (MR-PRESSO) test was applied to evaluate and adjust for horizontal pleiotropy because it can identify horizontal pleiotropy, remove outliers, and test for significant differences in causal estimates before and after outlier correction (Bowden et al., 2018). Leave-one-out (LOO) analysis was conducted to assess whether the summary estimate was biased by high-influence points (Burgess et al., 2017). Cochran’s Q test was used to calculate heterogeneity in the MR results (*p* < 0.05 indicated significant heterogeneity) (Greco et al., 2015). MR-Steiger analysis was employed to examine the direction of potential causal relationships for each extracted SNP regarding exposure and outcome, confirming that the correct direction indicated the absence of reverse causality (Hemani et al., 2017). The Bonferroni correction was used to determine the significance of multiple tests at each feature level for the primary MR results. The number of bacteria under each attribute was determined as follows: phyla: 0.05/9 (5.56 × 10^−3^), classes: 0.05/16 (3.13 × 10^−3^), orders: 0.05/20 (2.5 × 10^−3^), families: 0.05/32 (1.56 × 10^−3^), and genera: 0.05/119 (4.20 × 10^−4^).

All analyses were conducted using the MR (0.9.0), MR-PRESSO (1.0), and TwoSampleMR (0.5.8) packages in R (version 4.3.1). Statistical significance was set at *p* < 0.05. A significant two-sided *p*-value was set at 0.05 for the global-level test.

## Results

### Selection of IVs

After applying stringent control measures for *p*-values, LD effects, palindromic sequences, and F-statistic calculations, we identified 2,126 SNPs from 196 gut microbiota taxa to be included as IVs in the final MR analysis (Supplementary Table S1).

### Causative connection between gut microbiota and OAB

**Figure 2:**
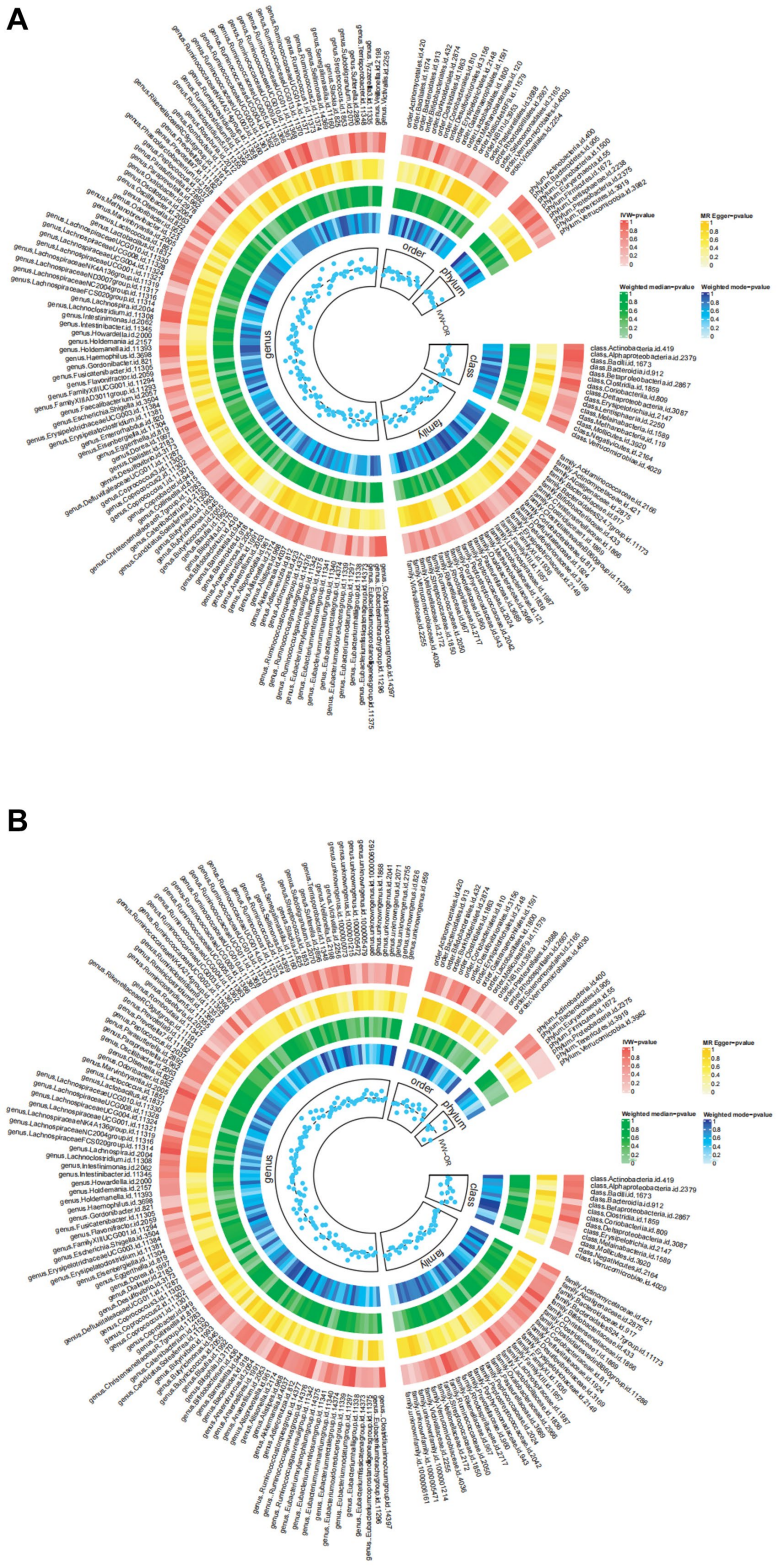
The detailed MR results for the associations between 196 bacterial taxa and OAB symptoms from the above four analysis methods. The estimations for the IVW MR-Egger, Weighted Median, and Weighted Mode are shown as circles from outer to inner. Gut microbiota is classified in genus, family, order, class and phylum. **(A)** The detailed MR results between gut microbiome and OAB; **(B)** The detailed MR results between gut microbiome and UI. IVW, inverse variance weighted; OAB, overactive bladder; UI, urinary incontinence.

**Figure 3:**
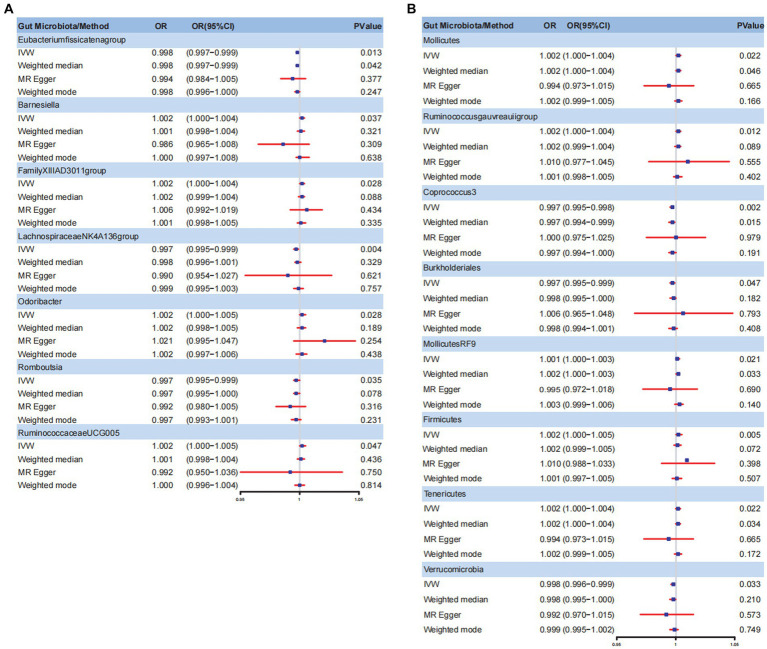
Forest plot for gut microbiome and OAB symptoms. **(A)** Forest plot for gut microbiome and OAB; **(B)** Forest plot for gut microbiome and UI. IVW, inverse variance weighted; OR, odds ratio; CI, confidence interval; OAB, overactive bladder; UI, urinary incontinence.

The Circus diagram depicts all results of the MR analysis for OAB (Figure 2A). Eventually, seven genera (97 SNPs) exhibited a significant causal relationship with OAB. Supplementary Table S2 presents the detailed GWAS information on the selected IVs. *Eubacteriumfissicatenagroup* (IVW: odds ratio [OR], 0.998; 95% confidence interval [CI]: 0.997–0.999; *p* = 0.013), genera *LachnospiraceaeNK4A136group* (IVW: OR, 0.997; 95% CI: 0.995–0.999; *p* = 0.004), and *Romboutsia* (IVW: OR, 0.997; 95% CI: 0.995–0.999; *p* = 0.035) were identified as protective factors against OAB. However, *Barnesiella* (IVW: OR, 1.002; 95% CI: 1.000–1.004; *p* = 0.037), *FamilyXIIIAD3011group* (IVW: OR, 1.002; 95% CI: 1.000–1.004; *p* = 0.028), *Odoribacter* (IVW: OR, 1.002; 95% CI: 1.000–1.005; *p* = 0.028), and *RuminococcaceaeUCG005* (IVW: OR, 1.002; 95% CI: 1.000–1.005; *p* = 0.047) were associated with an increased risk of OAB (Figure 3A). After applying stringent Bonferroni correction, no statistically significant causal relationships with OAB were observed. Table 1 presents the sensitivity analysis of the MR between the gut microbiota and OAB. The MR-PRESSO analysis results revealed no significant heterogeneity and outliers (global *P*_MR-PRESSO_ > 0.05), indicating no horizontal pleiotropy. No individual SNP significantly disturbed the overall effect of all exposures on OAB in the LOO analysis (Supplementary Figure S1). Analysis of all gut microbiota using MR-Egger regression identified no directional pleiotropy (Supplementary Figure S2). Furthermore, Cochran’s Q test results suggested the absence of heterogeneity, as all *p*-values were > 0.05. The MR-Steiger analysis confirmed the accuracy of the direction, reinforcing the robustness of causal effect estimates.

**Table 1:** Sensitivity analysis of MR between gut microbiota and OAB.

Level	Gut Microbiota	Horizontal pleiotropy				Heterogeneity		Steiger test
		MR-PRESSO global outlier test		MR-Egger regression		Q statistic	*p*-value	*p*-value
		Outlier	*p*-value	Intercept	*p*-value			
genus	Eubacteriumfissicatenagroup	NA	0.796	0.0004	0.501	2.534	0.771	1.77E-29
genus	Barnesiella	NA	0.673	0.0009	0.245	2.510	0.642	1.77E-23
genus	FamilyXIIIAD3011group	NA	0.944	−0.0002	0.611	1.699	0.888	1.72E-33
genus	LachnospiraceaeNK4A136group	NA	0.384	0.0003	0.724	8.723	0.366	2.95E-42
genus	Odoribacter	NA	0.335	−0.0010	0.305	2.135	0.544	3.64E-23
genus	Romboutsia	NA	0.423	0.0003	0.470	5.477	0.360	4.21E-28
genus	RuminococcaceaeUCG005	NA	0.362	0.0006	0.669	6.690	0.244	7.60E-36

OAB, overactive bladder; MR, Mendelian randomization; NA, not available.

### Causative connection between gut microbiota and UI

Figure 2B summarizes the results of the four analysis methods for the gut microbiota and UI. Ultimately, eight gut microbiota (120 SNPs) with a significance level of <0.05 were identified, comprising three phyla, one class, two orders, and two genera. Supplementary Table S3 provides details of the instrumental SNPs. A higher abundance of genus *Coprococcus3* (IVW: OR, 0.997; 95% CI: 0.995–0.998; *p* = 0.002), order Burkholderiales (IVW: OR, 0.997; 95% CI: 0.995–0.999; *p* = 0.047), and phylum Verrucomicrobia (IVW: OR, 0.998; 95% CI: 0.996–0.999; *p* = 0.033) predicted a lower risk of UI. However, class Mollicutes (IVW: OR, 1.002; 95% CI: 1.000–1.004; *p* = 0.022), genus *Ruminococcus gauvreauii* group (IVW: OR, 1.002; 95% CI: 1.000–1.004; *p* = 0.012), order MollicutesRF9 (IVW: OR, 1.001; 95% CI: 1.000–1.003; *p* = 0.021), and phylum Firmicutes (IVW: OR, 1.002; 95% CI: 1.000–1.005; *p* = 0.002) and Tenericutes (IVW: OR, 1.002; 95% CI: 1.000–1.004; *p* = 0.022) were positively correlated with UI risk (Figure 3B). The results of the Bonferroni-corrected test indicated that the phylum Firmicutes still exhibited a significant causal relationship with UI; however, no significant associations were observed for the remaining seven gut microbiota. The MR-PRESSO analysis and Cochran’s Q test indicated the absence of horizontal pleiotropy and heterogeneity. We detected no SNP outlier using LOO analysis (Supplementary Figure S3). The MR-Egger regression intercept was close to zero (*p* > 0.05), indicating a lack of directional pleiotropy (Supplementary Figure S4). The MR-Steiger analysis indicated no reverse causal relationships. In summary, the sensitivity analysis results indicated that the MR analysis results were robust and reliable (Table 2).

**Table 2:** Sensitivity analysis of MR between gut microbiota and UI.

Level	Gut microbiota	Horizontal pleiotropy				Heterogeneity		Steiger test
		MR-PRESSO global outlier test		MR-Egger regression		Q statistic	*p*-value	*p*-value
		Outlier	*p*-value	Intercept	*p*-value			
Class	Mollicutes	NA	0.814	0.0005	0.550	0.963	0.810	2.81E-19
Genus	Ruminococcusgauvreauiigroup	NA	0.246	−0.0005	0.643	8.416	0.209	4.82E-32
Genus	Coprococcus3	NA	0.876	−0.0001	0.807	1.169	0.947	3.09E-31
Order	Burkholderiales	NA	0.943	−0.0004	0.721	0.824	0.935	3.62E-22
Order	MollicutesRF9	NA	0.482	0.0004	0.592	6.131	0.408	1.34E-34
Phylum	Firmicutes	NA	0.169	−0.0003	0.530	5.320	0.377	1.59E-34
Phylum	Tenericutes	NA	0.842	0.0005	0.550	0.963	0.810	2.81E-19
Phylum	Verrucomicrobia	NA	0.447	0.0003	0.677	4.045	0.399	9.28E-23

UI, urinary incontinence; MR, Mendelian randomization; NA, not available.

## Discussion

To our knowledge, this is the first study to use MR analysis to investigate the genetically predicted causal relationship between the gut microbiota and OAB symptoms. Extensive research has investigated the relationship between the urinary tract microbiota and OAB symptoms. Researchers have explored the differences in the urine microbiota between individuals with OAB and the general population using high-throughput 16S ribosomal RNA (rRNA) gene sequencing to detect bacterial DNA in urine samples (Wolfe and Brubaker, 2019). The results revealed that patients with OAB exhibited significantly reduced microbial diversity compared to the control group (Hilt et al., 2014; Pohl et al., 2020; Okamoto et al., 2021). Changes in specific bacterial species in the urine, such as a reduction in *Lactobacillus* or an increase in *Escherichia coli*, may also worsen OAB symptoms (Karstens et al., 2016). Approximately 64% of the bacterial species in the urinary microbiota overlap with those found in the intestine, suggesting an intestinal origin (Holland et al., 2020). The link between gut microbiota and OAB symptoms is increasingly attracting the interest of researchers. Okamoto et al. (2021) conducted a cross-sectional survey and found that the relative abundance of *Bifidobacterium* was significantly reduced in the OAB population with daily urgency, while the relative abundance of *Faecalibacterium* was significantly higher in this group (Okamoto et al., 2021). The results of a three-year longitudinal study conducted by Okuyama et al. in Japan revealed that the genus *Streptococcus* (OR: 1.05, *p* = 0.029) is an independent risk factor for UI progression (Okuyama et al., 2022). Further research is needed to evaluate the causal relationship between the gut microbiota and OAB symptoms. This study employed new large-scale GWAS data and gene prediction methods to elucidate the relationship between specific gut microbiota and the progression of OAB symptoms. Consequently, the findings are more robust and offer reliable causal explanations, which could inform future treatments for OAB symptoms using targeted biological therapies.

This study identified 15 specific bacteria associated with OAB symptoms. Phylum Firmicutes still maintained a strong correlation after the Bonferroni-corrected test. Firmicutes is a phylum of bacteria characterized by thick cell walls primarily composed of peptidoglycans, reflected by its name “Firmicutes.” This phylum encompasses various bacteria with varying morphological and physiological traits. Previous studies have demonstrated that alterations in the proportion of Firmicutes and Bacteroidetes within the gut microbiota are closely associated with obesity (Ridaura et al., 2013). An increase in Firmicutes may contribute to obesity by enhancing energy absorption from food. Additionally, there was a correlation between obesity and OAB symptoms. Hsu et al. (2022) found that infusing fatty acid-derived prostaglandins into the bladder can induce uninhibited detrusor muscle contractions in animals and humans (Hsu et al., 2022). Moreover, they observed that serum nerve growth factor levels were positively correlated with obesity and other inflammatory markers in humans. According to the neurogenic theory of OAB, this condition may involve the local transfer of abnormal C-fiber activity to A-delta afferent fibers, typically involved in normal urination, leading to pathological states (Bhide et al., 2013). This may be the mechanism by which Firmicutes influence bladder function through metabolic pathways. *Streptococcus* is a genus belonging to the phylum Firmicutes. Okuyama et al. (2022) observed a significant increase in the genus *Streptococcus* in the progression of UI (*p* < 0.001) (Okuyama et al., 2022). Their additional analysis revealed that the relative abundance of this genus was higher in patients with irritable bowel syndrome (IBS) than in patients without IBS. The incidence of IBS was significantly higher in the UI progression group than in the control group (*p* = 0.025). Christianson et al. (2007) proposed that neural crosstalk between the parasympathetic and sympathetic nerves in the bladder and gastrointestinal tract may explain the association between OAB symptoms and IBS (Christianson et al., 2007). This indicates intestinal dysbiosis may influence bladder function via convergent sensory pathways between the intestine and bladder at the peripheral and spinal cord levels. The role of the gut-bladder axis in human lower urinary tract symptoms warrants increased attention (Schembri et al., 2022). A cross-sectional study in the Iwaki Health Promotion Project in Japan found that the genus *Faecalibacterium*, belonging to the phylum Firmicutes, is significantly more abundant in the OAB-daily incidence group (*p* = 0.006) (Okamoto et al., 2021). The short-chain fatty acids (SCFAs) produced by the genus *Faecalibacterium* generally benefit intestinal health. However, previous studies have indicated that excessive SCFAs can adversely affect human health (Tana et al., 2010). Kashyap et al. (2018) found that administration of butyric acid resulted in an increase in brain-derived neurotrophic factor in the urine of a rat experimental model, ultimately leading to enhanced bladder detrusor activity (Kashyap et al., 2018). Genera *Ruminococcus gauvreauii* group (IVW: OR, 1.002; 95% CI: 1.000–1.004; *p* = 0.012) and *RuminococcaceaeUCG005* (IVW: OR, 1.002; 95% CI: 1.000–1.005; *p* = 0.047), belonging to the phylum Firmicutes, were identified as risk factors for OAB symptoms in our research, although its significance diminished after Bonferroni correction. *Lactobacillus*, a main species within the phylum Firmicutes, has been predominantly studied for its role in the urine (Li K. et al., 2022). Revisited evidence in 2019 highlights its important regulatory and protective functions in the lower urinary tract (Antunes-Lopes and Cruz, 2019). However, research on how *Lactobacillus* in the digestive tract influences bladder function is currently limited and needs further exploration.

The Bonferroni-corrected test has the possibility of false-negative results. Although many gut microbiota have lost their correlation with OAB symptoms after correction, some findings still suggest potential relationships relevant to our research results. In our study, *Odoribacter* (belonging to the phylum Bacteroidetes) significantly correlated with OAB symptoms. Liu et al. (2024) discovered that genus *Odoribacter* in the gut remained significantly associated with chronic prostatitis even after false discovery rate correction (OR:1.43; 95% CI: 1.05–1.94) (Liu et al., 2024). Activation of the vagus nerve and regulation of the immune system by the gut microbiota may play a crucial role. However, whether this regulation affects chronic inflammation and the bladder’s function remains unclear. Our findings suggest that the *Barnesiella* genus, a significant member of the Bacteroidetes phylum, is a risk factor for OAB symptoms. A previous study identified a high abundance of the *Barnesiella* genus in the tumor mucosal microbiota, suggesting it may influence bladder pathology through unknown mechanisms (Parra-Grande et al., 2021). Therefore, further research is needed to explore the impact and mechanisms of the Bacteroidetes phylum in the gut on bladder pathology. The phylum Verrucomimicrobia was a protective factor against OAB symptoms in our research (IVW: OR, 0.998; 95% CI: 0.996–0.999; *p* = 0.033). *Akkermansia muciniphila* is an important member of the phylum Verrucomicrobia. Damage to intestinal epithelial integrity causes Gram-negative lipopolysaccharide accumulation in the serum, causing metabolic endotoxemia and systemic inflammatory responses that may affect bladder contraction (Cani et al., 2008). Reunanen et al. (2015) found that *Akkermansia muciniphila* binds to mucin layer adhesion proteins and undifferentiated Caco-2 cells, participating in the competitive exclusion of pathogenic organisms at the injury site (Reunanen et al., 2015). Following epithelial damage, it enhances the establishment of newly formed intestinal cell monolayers, significantly reducing lipopolysaccharide accumulation in the serum and the consequent inflammatory responses. These results have uncleared clinical guidance due to the disappearance of correlation after the Bonferroni-corrected test; however, they provide a theoretical basis for future research on gut microbiota and OAB symptoms.

Diagnosing and treating lower urinary tract diseases using gut microbiota remains in the preliminary research stages. Thus, there are several hypotheses regarding the mechanism of the impact of the gut microbiota on OAB. There is bidirectional communication between the gut and the central nervous system, known as the gut-brain axis. The gut microbiota may regulate the nervous system through various mechanisms, such as the production of neuroactive metabolites, modulation of the immune system, and activation of the vagus nerve, ultimately directly affecting the bladder (Cryan and Dinan, 2012). Research has discovered that gut microbiota can also contribute to the development of certain mental disorders by influencing early neural development mediated by SCFAs, inducing intestinal inflammation, and affecting metabolic and endocrine pathways (Li et al., 2023). These mental disorders may be closely related to the OAB symptoms (Smith et al., 2024). Funada et al. (2018) discovered a positive correlation between environmental factors and OAB symptoms, including depression, in male patients (Funada et al., 2018). Jung et al. (2023) also found that the urinary bacterial composition differs between patients with OAB and depression or anxiety and those without mental disorders (Jung et al., 2023). The direct or indirect effects of the gut-brain axis may influence bladder function changes; similarly, the gut-bladder axis is gradually gaining attention in research. Research suggests that antibiotic treatment can induce gut dysbiosis and increase the susceptibility to recurrent urinary tract infections. Dysbiosis is characterized by reduced microbial diversity, relative abundance of butyrate-producing microorganisms, and elevated plasma eotaxin-1 levels. These changes may represent a potential mechanism by which the gut-bladder axis regulates urinary tract infections (Schembri et al., 2022). A recent study in patients with UUI indicated the existence of a gut-bladder axis (Okamoto et al., 2021). Further research is needed to explore the complex signaling network between the pathophysiological changes in the gut and bladder, providing a foundation for future biologically targeted therapies for OAB.

It is essential to recognize that our study had certain limitations. First, the GWAS data related to gut microbiota were obtained from participants of diverse racial backgrounds, while the summary statistics for GWAS of OAB symptoms were derived solely from individuals of European descent. Second, although the identified SNPs are associated with gut microbiota composition, elucidating their specific links to individual OAB symptoms and their overall effects is more challenging. This complexity arises from the diverse roles that gut microbiota plays in health and disease. Third, direct methods were not employed to validate the second and third MR assumptions; any breach of these assumptions could lead to biased MR estimates. Future research should address these shortcomings and limitations.

In summary, we used MR analysis to identify gut microbiota associated with OAB symptoms from a genetic perspective. We identified 14 nominal causal relationships and one strong causal relationship, demonstrating a positive correlation between the Firmicutes phylum and OAB symptoms. It provided new avenues to identify biomarkers and therapeutic targets for patients with OAB symptoms, enhancing the overall quality of life for patients suffering from OAB symptoms.

## Data Availability

The datasets presented in this study can be found in online repositories. The names of the repository/repositories and accession number(s) can be found in the article/Supplementary material.
