# Supplementary material for: Causal effects of gut microbiota on risk of overactive bladder symptoms: a two-sample Mendelian randomization study
Source: Front Microbiol. 2024 Aug 23;15:1459634. doi: 10.3389/fmicb.2024.1459634 (PMC11380132; doi:10.3389/fmicb.2024.1459634)
Supplement: Supplementary file 1 [file Data_Sheet_1.zip › Captions.DOCX]

Supplementary Material

**
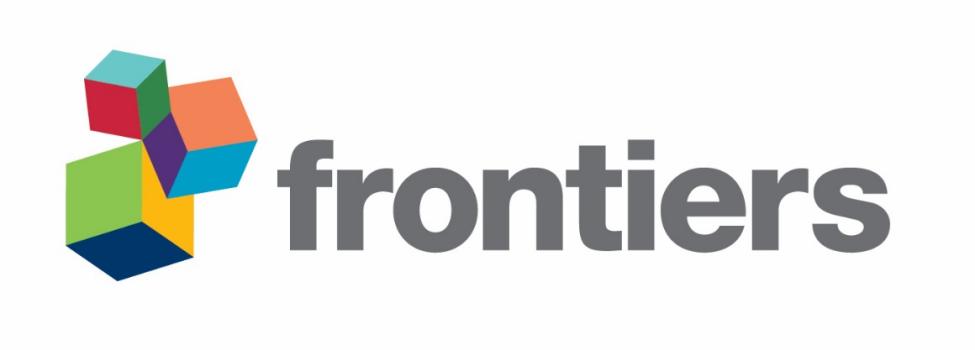
**

**Figure S1.** Visual image of Leave one out detection showing the Mendelian randomiza**tio**n effect of gut microbiome on OAB.

**Figure S2.** Scatter plot showing the Mendelian randomization effect of gut microbiome on OAB.

**Figure S3.** Visual image of Leave one out detection showing the Mendelian randomiza**tio**n effect of gut microbiome on UI.

**Figure S4.** Scatter plot showing the Mendelian randomization effect of gut microbiome on UI.

**Table S1.** Instrument variables for gut microbiota taxa enrolled in this Mendelian randomization study.

**Table S2.** List of instrumental SNPs of the causal gut microbiota taxa on Overactive Bladder.

**Table S3.** Table S3. List of instrumental SNPs of the causal gut microbiota taxa on Urinary Incontinence.
