# Supplementary material for: Causal effects of gut microbiota on risk of overactive bladder symptoms: a two-sample Mendelian randomization study
Source: Front Microbiol. 2024 Aug 23;15:1459634. doi: 10.3389/fmicb.2024.1459634 (PMC11380132; doi:10.3389/fmicb.2024.1459634)
Supplement: Supplementary file 1 [file Data_Sheet_1.zip › Figure S1.PDF]

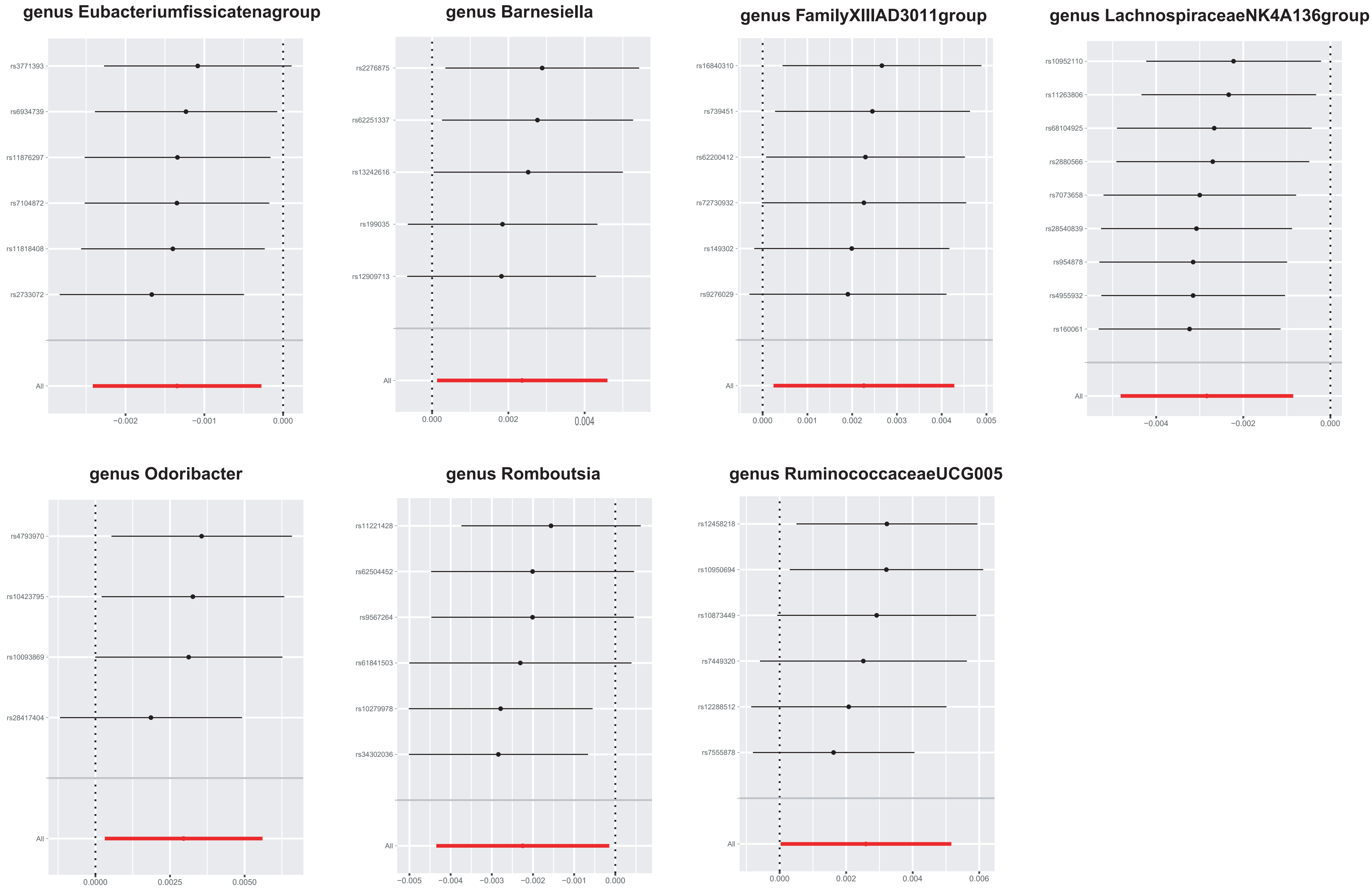
genus Eubacteriumfissicatenagroup
rs3771393
rs6934739
rs11876297
rs7104872
rs11818408
rs2733072
All
−0.002
−0.001
0.000
genus Barnesiella
rs2276875
rs62251337
rs13242616
rs199035
rs12909713
All
0.000
0.002
0.004
genus FamilyXIIIAD3011group
rs16840310
rs739451
rs62200412
rs72730932
rs149302
rs9276029
All
0.000
0.001
0.002
0.003
0.004
0.005
genus LachnospiraceaeNK4A136group
rs10952110
rs11263806
rs68104925
rs2880566
rs7073658
rs28540839
rs954878
rs4955932
rs160061
All
−0.004
−0.002
0.000
genus Odoribacter
rs4793970
rs10423795
rs10093869
rs28417404
All
0.0000
0.0025
0.0050
genus Romboutsia
rs11221428
rs62504452
rs9567264
rs61841503
rs10279978
rs34302036
All
−0.005
−0.004
−0.003
−0.002
−0.001
0.000
genus RuminococcaceaeUCG005
rs12458218
rs10950694
rs10873449
rs7449320
rs12288512
rs7555878
All
0.000
0.002
0.004
0.006
