# Supplementary material for: Causal effects of gut microbiota on risk of overactive bladder symptoms: a two-sample Mendelian randomization study
Source: Front Microbiol. 2024 Aug 23;15:1459634. doi: 10.3389/fmicb.2024.1459634 (PMC11380132; doi:10.3389/fmicb.2024.1459634)
Supplement: Supplementary file 1 [file Data_Sheet_1.zip › Figure S2.PDF]

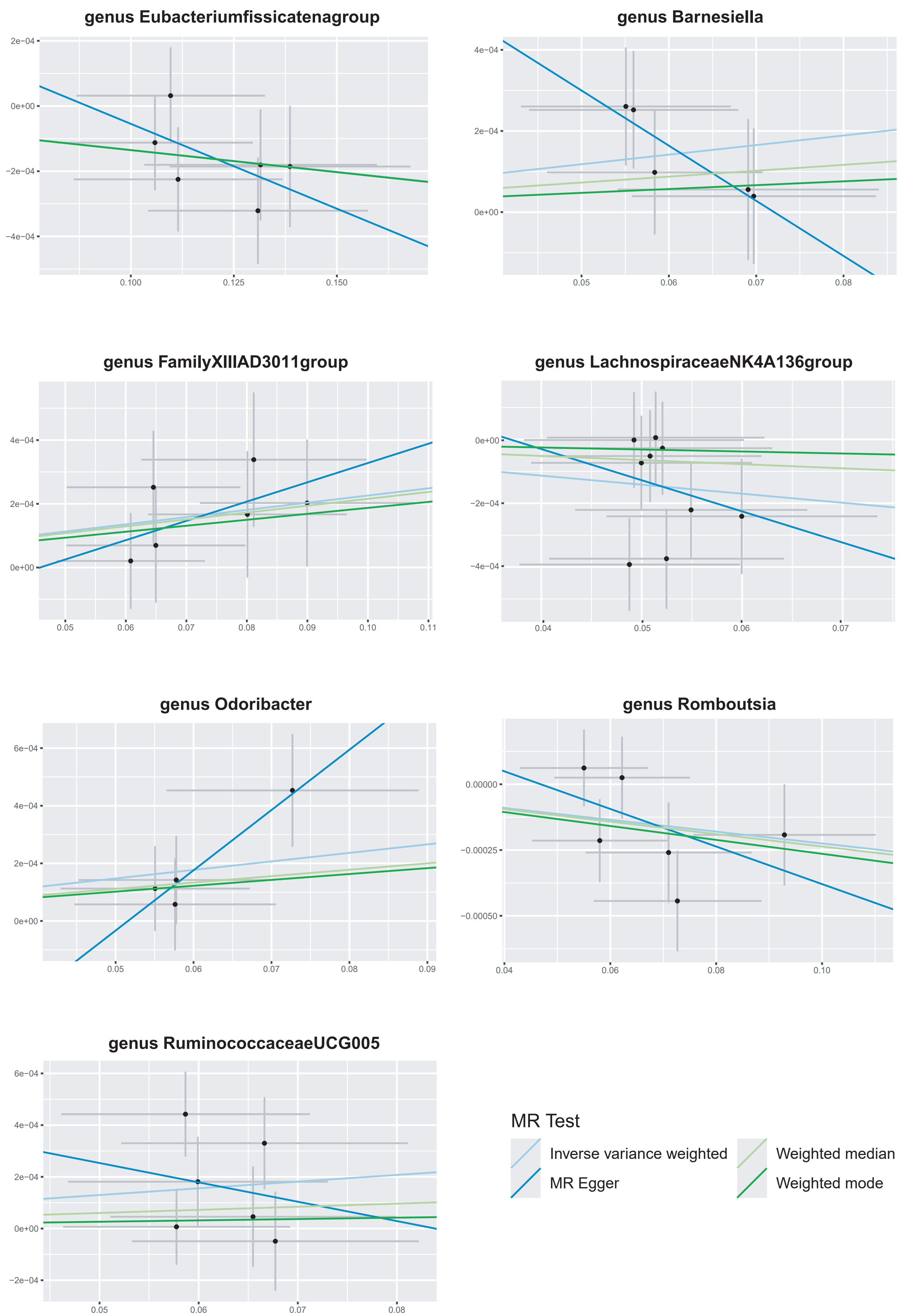

genus Eubacteriumfissicatenagroup
2e−04
0e+00
−2e−04
−4e−04
0.100
0.125
0.150
genus Barnesiella
4e−04
2e−04
0e+00
0.05
0.06
0.07
0.08
genus FamilyXIIIAD3011group
4e−04
2e−04
0e+00
0.05
0.06
0.07
0.08
0.09
0.10
0.11
genus LachnospiraceaeNK4A136group
0e+00
−2e−04
−4e−04
0.04
0.05
0.06
0.07
genus Odoribacter
6e−04
4e−04
2e−04
0e+00
0.05
0.06
0.07
0.08
0.09
genus Romboutsia
0.00000
−0.00025
−0.00050
0.04
0.06
0.08
0.10
genus RuminococcaceaeUCG005
6e−04
4e−04
2e−04
0e+00
−2e−04
0.05
0.06
0.07
0.08
MR Test
Inverse variance weighted
MR Egger
Weighted median
Weighted mode
