# Supplementary material for: Causal effects of gut microbiota on risk of overactive bladder symptoms: a two-sample Mendelian randomization study
Source: Front Microbiol. 2024 Aug 23;15:1459634. doi: 10.3389/fmicb.2024.1459634 (PMC11380132; doi:10.3389/fmicb.2024.1459634)
Supplement: Supplementary file 1 [file Data_Sheet_1.zip › Figure S3.PDF]

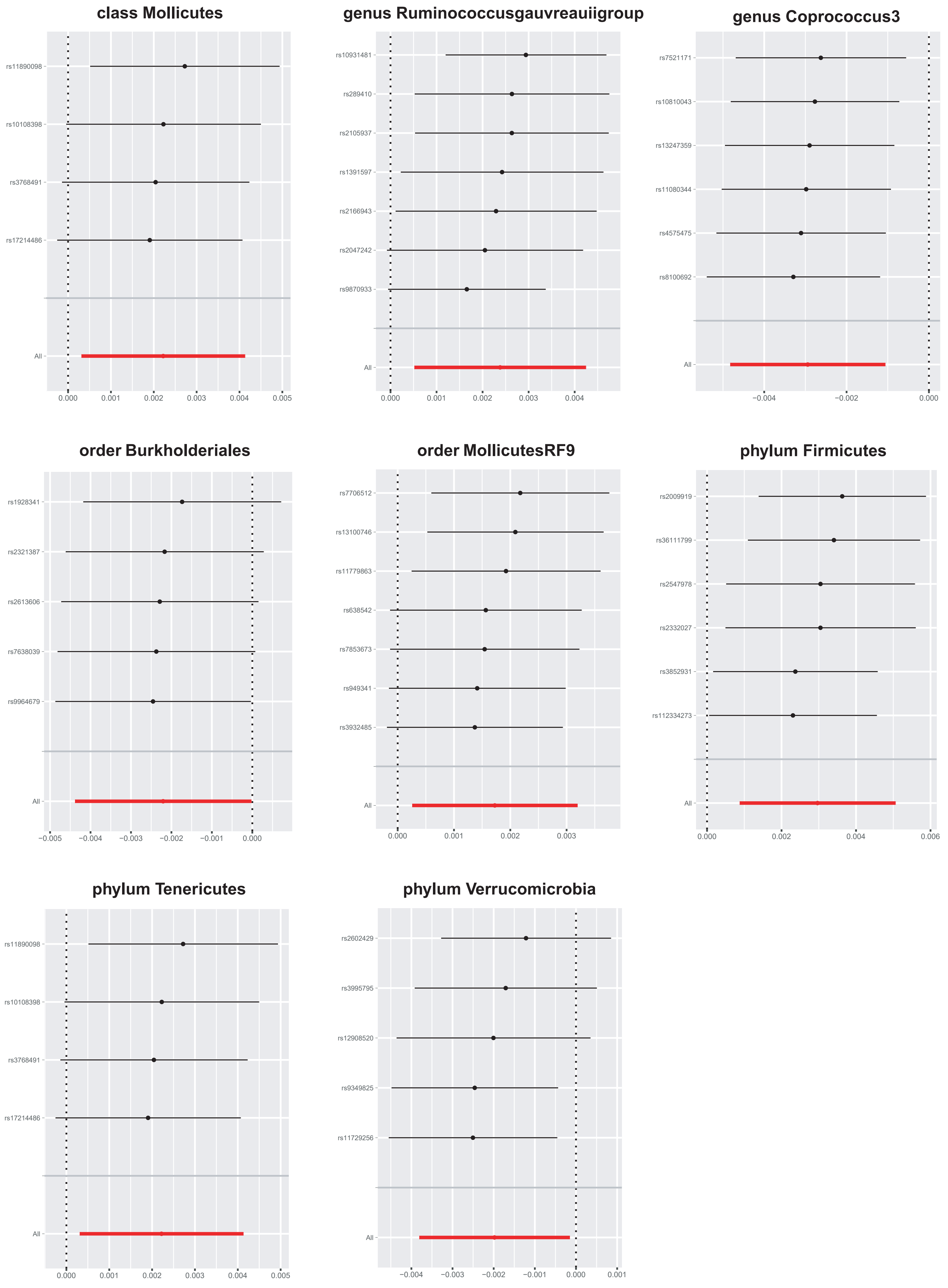
class Mollicutes
rs11890098
rs10108398
rs3768491
rs17214486
All
0.000 0.001 0.002 0.003 0.004 0.005
genus Ruminococcusgauvreauiigroup
rs10931481
rs289410
rs2105937
rs1391597
rs2166943
rs2047242
rs9870933
All
0.000 0.001 0.002 0.003 0.004
genus Coprococcus3
rs7521171
rs10810043
rs13247359
rs11080344
rs4575475
rs8100692
All
−0.004 −0.002 0.000
order Burkholderiales
rs1928341
rs2321387
rs2613606
rs7638039
rs9964679
All
−0.005 −0.004 −0.003 −0.002 −0.001 0.000
order MollicutesRF9
rs7706512
rs13100746
rs11779863
rs638542
rs7853673
rs949341
rs3932485
All
0.000 0.001 0.002 0.003
phylum Firmicutes
rs2009919
rs36111799
rs2547978
rs2332027
rs3852931
rs112334273
All
0.000 0.002 0.004 0.006
phylum Tenericutes
rs11890098
rs10108398
rs3768491
rs17214486
All
0.000 0.001 0.002 0.003 0.004 0.005
phylum Verrucomicrobia
rs2602429
rs3995795
rs12908520
rs9349825
rs11729256
All
−0.004 −0.003 −0.002 −0.001 0.000 0.001
