# Supplementary material for: Causal effects of gut microbiota on risk of overactive bladder symptoms: a two-sample Mendelian randomization study
Source: Front Microbiol. 2024 Aug 23;15:1459634. doi: 10.3389/fmicb.2024.1459634 (PMC11380132; doi:10.3389/fmicb.2024.1459634)
Supplement: Supplementary file 1 [file Data_Sheet_1.zip › Figure S4.PDF]

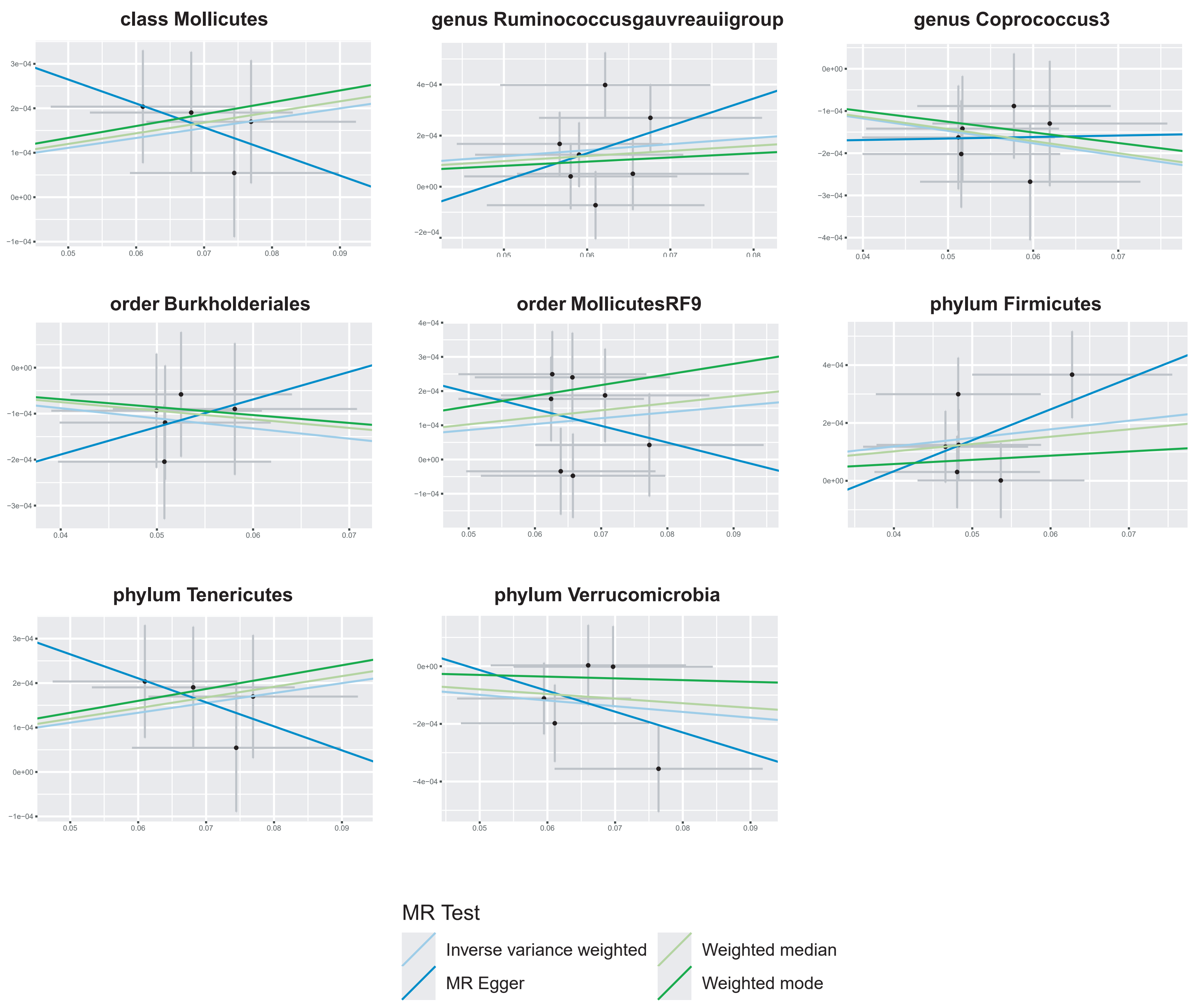
class Mollicutes
genus Ruminococcusgauvreauiigroup
genus Coprococcus3
order Burkholderiales
order MollicutesRF9
phylum Firmicutes
phylum Tenericutes
phylum Verrucomicrobia
MR Test
Inverse variance weighted
MR Egger
Weighted median
Weighted mode
