# Supplementary material for: Causal effects of gut microbiota on risk of overactive bladder symptoms: a two-sample Mendelian randomization study
Source: Front Microbiol. 2024 Aug 23;15:1459634. doi: 10.3389/fmicb.2024.1459634 (PMC11380132; doi:10.3389/fmicb.2024.1459634)
Supplement: Supplementary file 1 [file Data_Sheet_1.zip › STROBE-MR Checklist.docx]

**STROBE-MR checklist of recommended items to address in reports of Mendelian randomization studies**^1^ ^2^

| **Item No.** | **Section** | **Checklist item** | **Relevant text from manuscript** |
| --- | --- | --- | --- |
| 1 | **TITLE and ABSTRACT** | Indicate Mendelian randomization (MR) as the study’s design in the title and/or the abstract if that is a main purpose of the study | Causal effects of gut microbiota on risk of overactive bladder symptoms: A two-sample Mendelian randomization study |
|  | **INTRODUCTION** |  |  |
| 2 | **Background** | Explain the scientific background and rationale for the reported study. What is the exposure? Is a potential causal relationship between exposure and outcome plausible? Justify why MR is a helpful method to address the study question | Overactive bladder (OAB) is clinically diagnosed based on the presence of troublesome lower urinary tract symptoms. The International Continence Society defines OAB as the presence of "urinary urgency, usually accompanied by frequency and nocturia, with or without urgency urinary incontinence (UUI), in the absence of urinary tract infection or other obvious pathology"(Abrams et al. 2002). Previous studies conducted in different countries have reported OAB prevalence rates varying between 2.1% and 16.6%(Stewart et al. 2003, Homma, Yamaguchi og Hayashi 2005, Irwin et al. 2006). OAB symptoms significantly impact patients' quality of life, especially in patients with symptoms of urinary incontinence (UI); however, the precise mechanisms remain incompletely understood. A widely accepted theory explaining the pathophysiology of OAB involves detrusor overactivity(Brading 2006). Patients with OAB are treated with anti-cholinergic drugs and β3 agonists. However, some patients still experience symptoms despite treatment, highlighting that the mechanisms underlying OAB remain poorly understood(Gormley et al. 2012). Consequently, exploring the pathophysiological mechanisms leading to OAB formation is essential. This exploration is vital for developing innovative biological targeted therapy for pharmacological treatment. |
| 3 | **Objectives** | State specific objectives clearly, including pre-specified causal hypotheses (if any). State that MR is a method that, under specific assumptions, intends to estimate causal effects | This study used the latest available large-scale GWAS summary statistics for MR analysis to identify the potential causal relationship between gut microbiota and OAB symptoms, offering new perspectives for further mechanistic research and novel bio-targeted therapy. |
|  | **METHODS** |  |  |
| 4 | **Study design and data sources** | Present key elements of the study design early in the article. Consider including a table listing sources of data for all phases of the study. For each data source contributing to the analysis, describe the following: |  |
|  | a) | Setting: Describe the study design and the underlying population, if possible. Describe the setting, locations, and relevant dates, including periods of recruitment, exposure, follow-up, and data collection, when available. | In this study, two-sample MR analyses were conducted to determine the relationship between 196 gut microbiota and OAB symptoms, including UI. No additional ethical approval was needed because all the data used were published in a public database, and the original studies from which the summary statistics were derived had obtained proper ethical approval and informed consent. The analytic process was conducted per the Strengthening the Reporting of Observational Studies in Epidemiology using MR guidelines(Skrivankova et al. 2021). The following three assumptions must be satisfied for MR studies(Labrecque og Swanson 2018): (I) instrumental variables (IVs) are associated with the exposure of interest; (II) IVs are independent of any confounders in univariate MR; (III) IVs affect the outcome only through exposure. Figure 1 illustrates the study design and MR assumptions in this study. |
|  | b) | Participants: Give the eligibility criteria, and the sources and methods of selection of participants. Report the sample size, and whether any power or sample size calculations were carried out prior to the main analysis | All participants had similar genetic backgrounds, and each individual was of European ancestry. |
|  | c) | Describe measurement, quality control and selection of genetic variants | The following quality control steps were implemented to select the best IVs and ensure the validity and accuracy of the conclusions drawn regarding the causal relationship between the gut microbiome and OAB symptoms. Based on the correlation hypothesis, a threshold of P < 1 × 10−5 was used to screen for SNPs significantly associated with the gut microbiome, as they rarely reach genome-wide significance (P < 5 × 10−8)(Sanna et al. 2019). Additionally, the MR method requires that the selected IVs are not in linkage disequilibrium (LD) with each other. SNPs with an R2 < 0.001 and clustering distance of 10,000 kb were selected to retain independent SNPs and eliminate LD. Next, palindromic SNPs and SNPs absent in the outcome dataset were excluded to ensure that the effect of SNPs on exposure corresponded to the same allele as their effect on outcome(Hemani et al. 2018). Finally, to minimize the risk of weak instrument bias, the F-statistic for each gut microbiome-related SNP was calculated using the following formula: F = R2 × (n−k−1)/(k × (1−R2)). IVs with an F-statistic < 10 were defined as weak instruments and removed(Burgess og Thompson 2011, Levin et al. 2020). |
|  | d) | For each exposure, outcome, and other relevant variables, describe methods of assessment and diagnostic criteria for diseases | The MiBioGen consortium conducted a large-scale GWAS to examine the relationship between human genetic variation and the gut microbiome(Kurilshikov et al. 2021). This comprehensive study integrated the gut microbiome data from 18,340 participants across 24 cohorts from the United States, Canada, Israel, Germany, Sweden, Finland, and the United Kingdom, with the majority being of European descent (n = 13,266). In this study, data from the MiBioGen consortium encompassing 196 gut microbiome taxa (9 phyla, 16 classes, 20 orders, 33 families, and 119 genera) were used as exposure variables, excluding 15 unknown transgenic taxa (3 unknown families and 12 unknown genera).  The SNPs associated with OAB and UI were downloaded from the IEU OpenGWAS project database. For OAB, the phenotype "Bladder: Calcified/Contracted/Overactive" (GWAS ID: ukb-b-373)(Zhang et al. 2024), with a sample size of 463,010 and 9,851,867 SNPs, was selected. The summary data for UI were derived from the GWAS phenotype "Urinary frequency/Incontinence" (GWAS ID: ukb-b-8517)(Zhang et al. 2024), with a sample size of 462,933 and 9,851,867 SNPs. Notably, both databases were produced using a GWAS pipeline with phesant-derived variables from the UK Biobank. All participants had similar genetic backgrounds, and each individual was of European ancestry. |
|  | e) | Provide details of ethics committee approval and participant informed consent, if relevant | No additional ethical approval was needed because all the data used were published in a public database, and the original studies from which the summary statistics were derived had obtained proper ethical approval and informed consent. |
| 5 | **Assumptions** | Explicitly state the three core IV assumptions for the main analysis (relevance, independence and exclusion restriction) as well assumptions for any additional or sensitivity analysis | (I) instrumental variables (IVs) are associated with the exposure of interest; (II) IVs are independent of any confounders in univariate MR; (III) IVs affect the outcome only through exposure. Figure 1 illustrates the study design and MR assumptions in this study. |
| 6 | **Statistical methods: main analysis** | Describe statistical methods and statistics used |  |
|  | a) | Describe how quantitative variables were handled in the analyses (i.e., scale, units, model) | The following quality control steps were implemented to select the best IVs and ensure the validity and accuracy of the conclusions drawn regarding the causal relationship between the gut microbiome and OAB symptoms. Based on the correlation hypothesis, a threshold of P < 1 × 10−5 was used to screen for SNPs significantly associated with the gut microbiome, as they rarely reach genome-wide significance (P < 5 × 10−8)(Sanna et al. 2019). Additionally, the MR method requires that the selected IVs are not in linkage disequilibrium (LD) with each other. SNPs with an R2 < 0.001 and clustering distance of 10,000 kb were selected to retain independent SNPs and eliminate LD. Next, palindromic SNPs and SNPs absent in the outcome dataset were excluded to ensure that the effect of SNPs on exposure corresponded to the same allele as their effect on outcome(Hemani et al. 2018). Finally, to minimize the risk of weak instrument bias, the F-statistic for each gut microbiome-related SNP was calculated using the following formula: F = R2 × (n−k−1)/(k × (1−R2)). IVs with an F-statistic < 10 were defined as weak instruments and removed(Burgess og Thompson 2011, Levin et al. 2020). |
|  | b) | Describe how genetic variants were handled in the analyses and, if applicable, how their weights were selected | Four main methods were used in the MR analysis: inverse-variance weighted (IVW), weighted mode, MR-Egger regression, and weighted median. Among these, IVW was the primary assessment method, assuming that IVs affect the outcome solely through the exposure of interest and do not impact any other pathways(Bowden, Davey Smith og Burgess 2015). |
|  | c) | Describe the MR estimator (e.g. two-stage least squares, Wald ratio) and related statistics. Detail the included covariates and, in case of two-sample MR, whether the same covariate set was used for adjustment in the two samples | A range of methods were performed for sensitivity analysis. The MR-Egger regression intercept was used to estimate directional pleiotropy (P < 0.05 indicated directional pleiotropy)(Bowden, Davey Smith og Burgess 2015). The MR-Pleiotropy Residual Sum and Outlier (MR-PRESSO) test was applied to evaluate and adjust for horizontal pleiotropy because it can identify horizontal pleiotropy, remove outliers, and test for significant differences in causal estimates before and after outlier correction(Bowden et al. 2018). Leave-one-out (LOO) analysis was conducted to assess whether the summary estimate was biased by high-influence points(Burgess et al. 2017). Cochran’s Q test was used to calculate heterogeneity in the MR results (P < 0.05 indicated significant heterogeneity)(Greco et al. 2015). MR-Steiger analysis was employed to examine the direction of potential causal relationships for each extracted SNP regarding exposure and outcome, confirming that the correct direction indicated the absence of reverse causality(Hemani, Tilling og Davey Smith 2017). The Bonferroni correction was used to determine the significance of multiple tests at each feature level for the primary MR results. The number of bacteria under each attribute was determined as follows: phyla: 0.05/9 (5.56 × 10−3), classes: 0.05/16 (3.13 × 10−3), orders: 0.05/20 (2.5 × 10−3), families: 0.05/32 (1.56 × 10−3), and genera: 0.05/119 (4.20 × 10−4). |
|  | d) | Explain how missing data were addressed | There is no data loss in the data we use |
|  | e) | If applicable, indicate how multiple testing was addressed | Not applicable |
| 7 | **Assessment of assumptions** | Describe any methods or prior knowledge used to assess the assumptions or justify their validity | A range of methods were performed for sensitivity analysis. The MR-Egger regression intercept was used to estimate directional pleiotropy (P < 0.05 indicated directional pleiotropy)(Bowden, Davey Smith og Burgess 2015). The MR-Pleiotropy Residual Sum and Outlier (MR-PRESSO) test was applied to evaluate and adjust for horizontal pleiotropy because it can identify horizontal pleiotropy, remove outliers, and test for significant differences in causal estimates before and after outlier correction(Bowden et al. 2018). Leave-one-out (LOO) analysis was conducted to assess whether the summary estimate was biased by high-influence points(Burgess et al. 2017). Cochran’s Q test was used to calculate heterogeneity in the MR results (P < 0.05 indicated significant heterogeneity)(Greco et al. 2015). MR-Steiger analysis was employed to examine the direction of potential causal relationships for each extracted SNP regarding exposure and outcome, confirming that the correct direction indicated the absence of reverse causality(Hemani, Tilling og Davey Smith 2017). The Bonferroni correction was used to determine the significance of multiple tests at each feature level for the primary MR results. The number of bacteria under each attribute was determined as follows: phyla: 0.05/9 (5.56 × 10−3), classes: 0.05/16 (3.13 × 10−3), orders: 0.05/20 (2.5 × 10−3), families: 0.05/32 (1.56 × 10−3), and genera: 0.05/119 (4.20 × 10−4). |
| 8 | **Sensitivity analyses and additional analyses** | Describe any sensitivity analyses or additional analyses performed (e.g. comparison of effect estimates from different approaches, independent replication, bias analytic techniques, validation of instruments, simulations) | A range of methods were performed for sensitivity analysis. The MR-Egger regression intercept was used to estimate directional pleiotropy (P < 0.05 indicated directional pleiotropy)(Bowden, Davey Smith og Burgess 2015). The MR-Pleiotropy Residual Sum and Outlier (MR-PRESSO) test was applied to evaluate and adjust for horizontal pleiotropy because it can identify horizontal pleiotropy, remove outliers, and test for significant differences in causal estimates before and after outlier correction(Bowden et al. 2018). Leave-one-out (LOO) analysis was conducted to assess whether the summary estimate was biased by high-influence points(Burgess et al. 2017). Cochran’s Q test was used to calculate heterogeneity in the MR results (P < 0.05 indicated significant heterogeneity)(Greco et al. 2015). MR-Steiger analysis was employed to examine the direction of potential causal relationships for each extracted SNP regarding exposure and outcome, confirming that the correct direction indicated the absence of reverse causality(Hemani, Tilling og Davey Smith 2017). The Bonferroni correction was used to determine the significance of multiple tests at each feature level for the primary MR results. The number of bacteria under each attribute was determined as follows: phyla: 0.05/9 (5.56 × 10−3), classes: 0.05/16 (3.13 × 10−3), orders: 0.05/20 (2.5 × 10−3), families: 0.05/32 (1.56 × 10−3), and genera: 0.05/119 (4.20 × 10−4). |
| 9 | **Software and pre-registration** |  |  |
|  | a) | Name statistical software and package(s), including version and settings used | All analyses were conducted using the MR (0.9.0), MR-PRESSO (1.0), and TwoSampleMR (0.5.8) packages in R (version 4.3.1). Statistical significance was set at P < 0.05. A significant two-sided P-value was set at 0.05 for the global-level test. |
|  | b) | State whether the study protocol and details were pre-registered (as well as when and where) | not |
|  | **RESULTS** |  |  |
| 10 | **Descriptive data** |  |  |
|  | a) | Report the numbers of individuals at each stage of included studies and reasons for exclusion. Consider use of a flow diagram | After applying stringent control measures for P-values, LD effects, palindromic sequences, and F-statistic calculations, we identified 2,126 SNPs from 196 gut microbiota taxa to be included as IVs in the final MR analysis (Table S1). |
|  | b) | Report summary statistics for phenotypic exposure(s), outcome(s), and other relevant variables (e.g. means, SDs, proportions) | Table S2 presents the detailed GWAS information on the selected IVs.  Table S3 provides details of the instrumental SNPs. |
|  | c) | If the data sources include meta-analyses of previous studies, provide the assessments of heterogeneity across these studies | Not applicable |
|  | d) | For two-sample MR:  i.  Provide justification of the similarity of the genetic variant-exposure associations between the exposure and outcome samples  ii.  Provide information on the number of individuals who overlap between the exposure and outcome studies | The Circus diagram depicts all results of the MR analysis for OAB (Figure 2A). Eventually, seven genera (97 SNPs) exhibited a significant causal relationship with OAB. Table S2 presents the detailed GWAS information on the selected IVs. Eubacteriumfissicatenagroup (IVW: odds ratio [OR], 0.998; 95% confidence interval [CI]: 0.997–0.999; P = 0.013), generaLachnospiraceaeNK4A136group (IVW: OR, 0.997; 95% CI: 0.995–0.999; P = 0.004), and Romboutsia (IVW: OR, 0.997; 95% CI: 0.995–0.999; P = 0.035) were identified as protective factors against OAB. However, Barnesiella (IVW: OR, 1.002; 95% CI: 1.000–1.004; P = 0.037), FamilyXIIIAD3011group (IVW: OR, 1.002; 95% CI: 1.000–1.004; P = 0.028), Odoribacter (IVW: OR, 1.002; 95% CI: 1.000–1.005; P = 0.028), and RuminococcaceaeUCG005 (IVW: OR, 1.002; 95% CI: 1.000–1.005; P = 0.047) were associated with an increased risk of OAB (Figure 3A). |
| 11 | **Main results** |  |  |
|  | a) | Report the associations between genetic variant and exposure, and between genetic variant and outcome, preferably on an interpretable scale | Figure 2B summarizes the results of the four analysis methods for the gut microbiota and UI. Ultimately, eight gut microbiota (120 SNPs) with a significance level of < 0.05 were identified, comprising three phyla, one class, two orders, and two genera. Table S3 provides details of the instrumental SNPs. A higher abundance of genus Coprococcus3 (IVW: OR, 0.997; 95% CI: 0.995–0.998; P = 0.002), order Burkholderiales (IVW: OR, 0.997; 95% CI: 0.995–0.999; P = 0.047), and phylum Verrucomicrobia (IVW: OR, 0.998; 95% CI: 0.996–0.999; P = 0.033) predicted a lower risk of UI. However, class Mollicutes (IVW: OR, 1.002; 95% CI: 1.000–1.004; P = 0.022), genus Ruminococcus gauvreauii group (IVW: OR, 1.002; 95% CI: 1.000–1.004; P = 0.012), order MollicutesRF9 (IVW: OR, 1.001; 95% CI: 1.000–1.003; P = 0.021), and phylum Firmicutes (IVW: OR, 1.002; 95% CI: 1.000–1.005; P = 0.002) and Tenericutes (IVW: OR, 1.002; 95% CI: 1.000–1.004; P = 0.022) were positively correlated with UI risk (Figure 3B). |
|  | b) | Report MR estimates of the relationship between exposure and outcome, and the measures of uncertainty from the MR analysis, on an interpretable scale, such as odds ratio or relative risk per SD difference | The Circus diagram depicts all results of the MR analysis for OAB (Figure 2A). Eventually, seven genera (97 SNPs) exhibited a significant causal relationship with OAB. Table S2 presents the detailed GWAS information on the selected IVs. Eubacteriumfissicatenagroup (IVW: odds ratio [OR], 0.998; 95% confidence interval [CI]: 0.997–0.999; P = 0.013), generaLachnospiraceaeNK4A136group (IVW: OR, 0.997; 95% CI: 0.995–0.999; P = 0.004), and Romboutsia (IVW: OR, 0.997; 95% CI: 0.995–0.999; P = 0.035) were identified as protective factors against OAB. However, Barnesiella (IVW: OR, 1.002; 95% CI: 1.000–1.004; P = 0.037), FamilyXIIIAD3011group (IVW: OR, 1.002; 95% CI: 1.000–1.004; P = 0.028), Odoribacter (IVW: OR, 1.002; 95% CI: 1.000–1.005; P = 0.028), and RuminococcaceaeUCG005 (IVW: OR, 1.002; 95% CI: 1.000–1.005; P = 0.047) were associated with an increased risk of OAB (Figure 3A). |
|  | c) | If relevant, consider translating estimates of relative risk into absolute risk for a meaningful time period | Figure 2B summarizes the results of the four analysis methods for the gut microbiota and UI. Ultimately, eight gut microbiota (120 SNPs) with a significance level of < 0.05 were identified, comprising three phyla, one class, two orders, and two genera. Table S3 provides details of the instrumental SNPs. A higher abundance of genus Coprococcus3 (IVW: OR, 0.997; 95% CI: 0.995–0.998; P = 0.002), order Burkholderiales (IVW: OR, 0.997; 95% CI: 0.995–0.999; P = 0.047), and phylum Verrucomicrobia (IVW: OR, 0.998; 95% CI: 0.996–0.999; P = 0.033) predicted a lower risk of UI. However, class Mollicutes (IVW: OR, 1.002; 95% CI: 1.000–1.004; P = 0.022), genus Ruminococcus gauvreauii group (IVW: OR, 1.002; 95% CI: 1.000–1.004; P = 0.012), order MollicutesRF9 (IVW: OR, 1.001; 95% CI: 1.000–1.003; P = 0.021), and phylum Firmicutes (IVW: OR, 1.002; 95% CI: 1.000–1.005; P = 0.002) and Tenericutes (IVW: OR, 1.002; 95% CI: 1.000–1.004; P = 0.022) were positively correlated with UI risk (Figure 3B). |
|  | d) | Consider plots to visualize results (e.g. forest plot, scatterplot of associations between genetic variants and outcome versus between genetic variants and exposure) | No individual SNP significantly disturbed the overall effect of all exposures on OAB in the LOO analysis (Figure S1). |
| 12 | **Assessment of assumptions** |  |  |
|  | a) | Report the assessment of the validity of the assumptions | The MR-PRESSO analysis results revealed no significant heterogeneity and outliers (global PMR-PRESSO > 0.05), indicating no horizontal pleiotropy. No individual SNP significantly disturbed the overall effect of all exposures on OAB in the LOO analysis (Figure S1). Analysis of all gut microbiota using MR-Egger regression identified no directional pleiotropy (Figure S2). Furthermore, Cochran’s Q test results suggested the absence of heterogeneity, as all P-values were > 0.05. The MR-Steiger analysis confirmed the accuracy of the direction, reinforcing the robustness of causal effect estimates. |
|  | b) | Report any additional statistics (e.g., assessments of heterogeneity across genetic variants, such as *I^2^*, Q statistic or E-value) | The results of the Bonferroni-corrected test indicated that the phylum Firmicutes still exhibited a significant causal relationship with UI; however, no significant associations were observed for the remaining seven gut microbiota. The MR-PRESSO analysis and Cochran’s Q test indicated the absence of horizontal pleiotropy and heterogeneity. We detected no SNP outlier using LOO analysis (Figure S3). The MR-Egger regression intercept was close to zero (P > 0.05), indicating a lack of directional pleiotropy Figure S4). (The MR-Steiger analysis indicated no reverse causal relationships. In summary, the sensitivity analysis results indicated that the MR analysis results were robust and reliable (Table 2). |
| 13 | **Sensitivity analyses and additional analyses** |  |  |
|  | a) | Report any sensitivity analyses to assess the robustness of the main results to violations of the assumptions | (The MR-Steiger analysis indicated no reverse causal relationships. In summary, the sensitivity analysis results indicated that the MR analysis results were robust and reliable (Table 2). |
|  | b) | Report results from other sensitivity analyses or additional analyses | The MR-Egger regression intercept was close to zero (P > 0.05), indicating a lack of directional pleiotropy Figure S4) |
|  | c) | Report any assessment of direction of causal relationship (e.g., bidirectional MR) | Analysis of all gut microbiota using MR-Egger regression identified no directional pleiotropy (Figure S2). Furthermore, Cochran’s Q test results suggested the absence of heterogeneity |
|  | d) | When relevant, report and compare with estimates from non-MR analyses | NA |
|  | e) | Consider additional plots to visualize results (e.g., leave-one-out analyses) | No individual SNP significantly disturbed the overall effect of all exposures on OAB in the LOO analysis (Figure S1). |
|  | **DISCUSSION** |  |  |
| 14 | **Key results** | Summarize key results with reference to study objectives | To our knowledge, this is the first study to use MR analysis to investigate the genetically predicted causal relationship between the gut microbiota and OAB symptoms. |
| 15 | **Limitations** | Discuss limitations of the study, taking into account the validity of the IV assumptions, other sources of potential bias, and imprecision. Discuss both direction and magnitude of any potential bias and any efforts to address them | It is essential to recognize that our study had certain limitations. First, the GWAS data related to gut microbiota were obtained from participants of diverse racial backgrounds, while the summary statistics for GWAS of OAB symptoms were derived solely from individuals of European descent. Second, although the identified SNPs are associated with gut microbiota composition, elucidating their specific links to individual OAB symptoms and their overall effects is more challenging. This complexity arises from the diverse roles that gut microbiota plays in health and disease. Third, direct methods were not employed to validate the second and third MR assumptions; any breach of these assumptions could lead to biased MR estimates. Future research should address these shortcomings and limitations. |
| 16 | **Interpretation** |  |  |
|  | a) | Meaning: Give a cautious overall interpretation of results in the context of their limitations and in comparison with other studies | Researchers have explored the differences in the urine microbiota between individuals with OAB and the general population using high-throughput 16S ribosomal RNA (rRNA) gene sequencing to detect bacterial DNA in urine samples(Wolfe og Brubaker 2019). The results revealed that patients with OAB exhibited significantly reduced microbial diversity compared to the control group(Hilt et al. 2014, Pohl et al. 2020, Okamoto et al. 2021). Changes in specific bacterial species in the urine, such as a reduction in Lactobacillus or an increase in Escherichia coli, may also worsen OAB symptoms(Karstens et al. 2016). Approximately 64% of the bacterial species in the urinary microbiota overlap with those found in the intestine, suggesting an intestinal origin. The link between gut microbiota and OAB symptoms is increasingly attracting the interest of researchers. Okamoto et al.(Okamoto et al. 2021) conducted a cross-sectional survey and found that the relative abundance of Bifidobacterium was significantly reduced in the OAB population with daily urgency, while the relative abundance of Faecalibacterium was significantly higher in this group. The results of a three-year longitudinal study conducted by Okuyama et al. in Japan revealed that the genus Streptococcus (OR: 1.05, P = 0.029) is an independent risk factor for UI progression(Okuyama et al. 2022). Further research is needed to evaluate the causal relationship between the gut microbiota and OAB symptoms. This study employed new large-scale GWAS data and gene prediction methods to elucidate the relationship between specific gut microbiota and the progression of OAB symptoms. Consequently, the findings are more robust and offer reliable causal explanations, which could inform future treatments for OAB symptoms using targeted biological therapies. |
|  | b) | Mechanism: Discuss underlying biological mechanisms that could drive a potential causal relationship between the investigated exposure and the outcome, and whether the gene-environment equivalence assumption is reasonable. Use causal language carefully, clarifying that IV estimates may provide causal effects only under certain assumptions | Diagnosing and treating lower urinary tract diseases using gut microbiota remains in the preliminary research stages. Thus, there are several hypotheses regarding the mechanism of the impact of the gut microbiota on OAB. There is bidirectional communication between the gut and the central nervous system, known as the gut-brain axis. The gut microbiota may regulate the nervous system through various mechanisms, such as the production of neuroactive metabolites, modulation of the immune system, and activation of the vagus nerve, ultimately directly affecting the bladder(Cryan og Dinan 2012). Research has discovered that gut microbiota can also contribute to the development of certain mental disorders by influencing early neural development mediated by SCFAs, inducing intestinal inflammation, and affecting metabolic and endocrine pathways(Li et al. 2023). These mental disorders may be closely related to the OAB symptoms(Smith et al. 2024). Funada et al.(Funada et al. 2018) discovered a positive correlation between environmental factors and OAB symptoms, including depression, in male patients. Jung et al.(Jung, Kim og Yang 2023) also found that the urinary bacterial composition differs between patients with OAB and depression or anxiety and those without mental disorders. The direct or indirect effects of the gut-brain axis may influence bladder function changes; similarly, the gut-bladder axis is gradually gaining attention in research. Research suggests that antibiotic treatment can induce gut dysbiosis and increase the susceptibility to recurrent urinary tract infections. Dysbiosis is characterized by reduced microbial diversity, relative abundance of butyrate-producing microorganisms, and elevated plasma eotaxin-1 levels. These changes may represent a potential mechanism by which the gut-bladder axis regulates urinary tract infections(Schembri, Nhu og Phan 2022). A recent study in patients with UUI indicated the existence of a gut-bladder axis(Okamoto et al. 2021). Further research is needed to explore the complex signaling network between the pathophysiological changes in the gut and bladder, providing a foundation for future biologically targeted therapies for OAB. |
|  | c) | Clinical relevance: Discuss whether the results have clinical or public policy relevance, and to what extent they inform effect sizes of possible interventions | The Bonferroni-corrected test has the possibility of false-negative results. Although many gut microbiota have lost their correlation with OAB symptoms after correction, some findings still suggest potential relationships relevant to our research results. In our study, Odoribacter (belonging to the phylum Bacteroidetes) significantly correlated with OAB symptoms. Liu et al.(Wei et al. 2023) discovered that genus Odoribacter in the gut remained significantly associated with chronic prostatitis even after false discovery rate correction (OR:1.43; 95% CI: 1.05–1.94). Activation of the vagus nerve and regulation of the immune system by the gut microbiota may play a crucial role. However, whether this regulation affects chronic inflammation and the bladder's function remains unclear. Our findings suggest that the Barnesiella genus, a significant member of the Bacteroidetes phylum, is a risk factor for OAB symptoms. A previous study identified a high abundance of the Barnesiella genus in the tumor mucosal microbiota, suggesting it may influence bladder pathology through unknown mechanisms(Parra-Grande et al. 2021). Therefore, further research is needed to explore the impact and mechanisms of the Bacteroidetes phylum in the gut on bladder pathology. The phylum Verrucomimicrobia was a protective factor against OAB symptoms in our research (IVW: OR, 0.998; 95% CI: 0.996–0.999; P = 0.033). Akkermansia muciniphila is an important member of the phylum Verrucomicrobia. Damage to intestinal epithelial integrity causes Gram-negative lipopolysaccharide accumulation in the serum, causing metabolic endotoxemia and systemic inflammatory responses that may affect bladder contraction(Cani et al. 2008). Reunanen et al.(Reunanen et al. 2015) found that Akkermansia muciniphila binds to mucin layer adhesion proteins and undifferentiated Caco-2 cells, participating in the competitive exclusion of pathogenic organisms at the injury site. Following epithelial damage, it enhances the establishment of newly formed intestinal cell monolayers, significantly reducing lipopolysaccharide accumulation in the serum and the consequent inflammatory responses. These results have uncleared clinical guidance due to the disappearance of correlation after the Bonferroni-corrected test; however, they provide a theoretical basis for future research on gut microbiota and OAB symptoms. |
| 17 | **Generalizability** | Discuss the generalizability of the study results (a) to other populations, (b) across other exposure periods/timings, and (c) across other levels of exposure | we used MR analysis to identify gut microbiota associated with OAB symptoms from a genetic perspective. We identified 14 nominal causal relationships and one strong causal relationship, demonstrating a positive correlation between the Firmicutes phylum and OAB symptoms, providing new avenues to identify biomarkers and therapeutic targets for patients with OAB.. |
|  | **OTHER INFORMATION** |  |  |
| 18 | **Funding** | Describe sources of funding and the role of funders in the present study and, if applicable, sources of funding for the databases and original study or studies on which the present study is based | Not applicable |
| 19 | **Data and data sharing** | Provide the data used to perform all analyses or report where and how the data can be accessed, and reference these sources in the article. Provide the statistical code needed to reproduce the results in the article, or report whether the code is publicly accessible and if so, where | The original contributions presented in the study are included in the article/Supplementary material, further inquiries can be directed to the corresponding author. |
| 20 | **Conflicts of Interest** | All authors should declare all potential conflicts of interest | The authors have no conflicts of interest to declare. |

This checklist is copyrighted by the Equator Network under the Creative Commons Attribution 3.0 Unported (CC BY 3.0) license.

1. Skrivankova VW, Richmond RC, Woolf BAR, Yarmolinsky J, Davies NM, Swanson SA, et al. Strengthening the Reporting of Observational Studies in Epidemiology using Mendelian Randomization (STROBE-MR) Statement. JAMA. 2021;under review.

2. Skrivankova VW, Richmond RC, Woolf BAR, Davies NM, Swanson SA, VanderWeele TJ, et al. Strengthening the Reporting of Observational Studies in Epidemiology using Mendelian Randomisation (STROBE-MR): Explanation and Elaboration. BMJ. 2021;375:n2233.
